# A 64-Year-Old Woman with Chest Pain, Limb Weakness, and Endometrial Cancer

**DOI:** 10.1155/2017/1891897

**Published:** 2017-03-08

**Authors:** Simon Ponthus, Omar Kherad, Nicole Petriccioli, Johannes Alexander Lobrinus, Pierre-André A. Guerne

**Affiliations:** ^1^Internal Medicine Department, Hôpital de la Tour, Geneva, Switzerland; ^2^Department of Pathology, Geneva University Hospitals, Geneva, Switzerland; ^3^Department of Rheumatology, Geneva University Hospitals, Geneva, Switzerland

## Abstract

Necrotizing autoimmune myopathy (NAM) is a rare subgroup of idiopathic inflammatory myopathies (IIM). This pathology usually affects proximal limb muscles and in some cases the myocardium. Patients usually display proximal limb weakness. Muscular biopsy is required to confirm the diagnosis. We report the case of a 64-year-old woman with an atypical first presentation of NAM, manifested by chest pain in the context of metastatic endometrial cancer. The diagnosis of NAM was however made when she returned a second time with proximal limb weakness. A treatment with prednisone was then initiated, to which rituximab was rapidly associated, beside a specific chemotherapy.

## 1. Background

NAM is part of the spectrum of the inflammatory myopathies [1]. It is characterized by muscle biopsy findings that include necrosis of muscle fibers with concomitant regeneration and low level inflammation, particularly mediated by macrophages [2]. At least 4 types of different etiologies of NAM are recognized: antisignal recognition particle (anti-SRP) antibody syndrome; a subgroup of statin-triggered autoimmune myositis, sometimes associated with anti-3-hydroxy-3methylglutaryl-coenzyme A reductase antibodies (anti-HMGCR); and paraneoplastic syndromes and viral infections, especially human immunodeficiency virus (HIV) [3]. Anti-SRP syndrome is a severe form of NAM characterized by a rapid progression and frequent myocardial involvement. Endometrial cancer related to NAM has not yet been described in the literature.

## 2. Case Presentation

A 64-year-old woman, known for a uterine clear-cell carcinoma (pT1aNOG3) and treated by hysterectomy two years ago, presented to the emergency room with chest pain. She had no cardiovascular risk factors except for her age. Troponins and creatine kinase (CK) levels returned elevated (1383 ng/L and 8808 U/L resp.); the electrocardiogram was normal. Even though no significant coronary lesion was detected on the cardiac catheterization, a diagnosis of non-ST elevation myocardial infarction (NSTEMI) was made. A treatment of atorvastatin 20 mg and aspirin 100 mg once a day was prescribed. During this hospital stay, a CT-scan revealed pulmonary and mediastinal masses. A biopsy confirmed metastases of endometrial origin and chemotherapy with Paclitaxel and Carboplatin was initiated. Two months later, her oncologist referred her for investigation of deterioration of health status. She complained about shoulder and thigh weakness over the previous few weeks, preventing her from climbing stairs and lifting objects. She also mentioned having difficulty swallowing (dysphagia). She did not describe paresthesia or any other neurological symptoms and no chest pain. Physical exam revealed global amyotrophy of limbs mostly proximal. Symmetrical severe paresis (M2/5) of all proximal muscles was observed, while strength of biceps and triceps was only slightly diminished (M4/M5) and conserved more distally (hands). No sensitive deficit was noticed; the rest of the neurological examination was normal. She had no noticeable cutaneous manifestation.

## 3. Investigations

**Figure 1:**
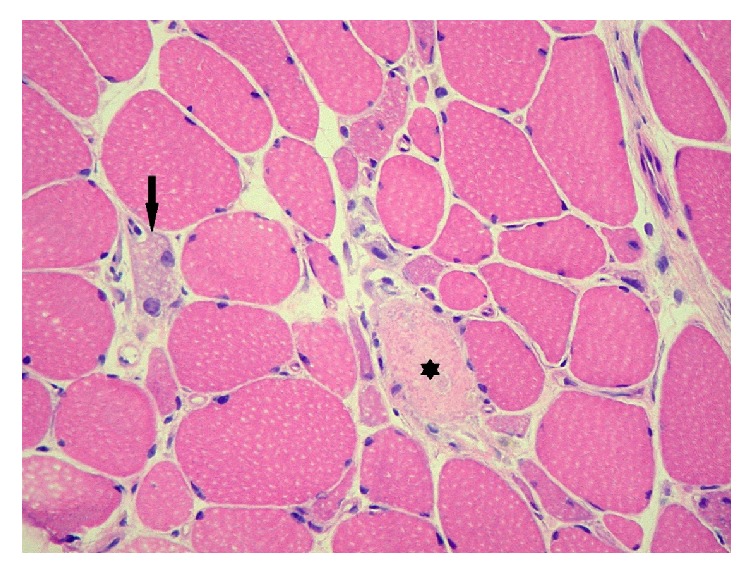
Muscle histology: frozen H&E stained section, showing a necrotic muscle fiber (asterisk), with marked fiber size variation and regeneration (arrow), without lymphocytic infiltration (original magnification 200x).

Laboratory findings (Table 1) showed elevated CKs (10176  U/L), Aldolase (67 U/L), ASAT (438 U/L), ALAT (327 U/L), and LDH (1187 U/L) consistent with possible cellular muscular lysis. Troponins were also high (1198 ng/L). Antiacetylcholine receptor (anti-AChR) and muscle-specific kinase (anti-MuSK) antibodies for myasthenia gravis were negative. Lambert-Eaton myasthenic syndrome antibodies (voltage gate calcium channel (VGCC) antibodies) were also negative. Cerebral and spinal MRI did not show any metastasis or other lesion consistent with the symptoms and signs. The electromyoneurography exam of the deltoid was consistent with a myopathic tracing. A needle biopsy was performed in the right quadriceps femoris muscle. Scattered necrotic muscle fibers, myophagia, and regeneration were observed. Furthermore, the muscle fibers showed marked size variation with few internal nuclei. Fibrosis was minimal and no significant lymphocytic inflammatory infiltration was observed (Figure 1). All of this was consistent with the diagnosis of NAM, which was confirmed by the finding of positive antisignal recognition particle (anti-SRP) antibodies in the serum.

## 4. Treatment

Prednisone 1 mg/kg once a day was given during two month followed by a progressive reduction. Rituximab was rapidly introduced (500 mg once a week during 4 weeks) followed by methotrexate (15 mg once a week). She continued specific chemotherapy, as treatment of neoplasia was necessary in this possibly paraneoplastic symptomatology [4]. She also benefited from physiotherapy as exercise seems to be effective in patients with IIM [5]. All treatments were well tolerated without major side effect.

## 5. Outcome and Follow-Up

Four months after the diagnosis and the beginning of treatments, we observed significant progress in her strength. She is now able to get up from her bed and walk without any help. Climbing stairs is still difficult but she also progressed in that point. Moving her shoulders and carrying objects is now easier. Rheumatologists of Geneva University Hospitals organized a follow-up.

## 6. Discussion

This case illustrates the complexity of NAM for which the etiological research can be challenging. This patient had endometrial cancer. Thus, the NAM could have been a paraneoplastic manifestation. Paraneoplastic NAM have been associated with gastrointestinal adenocarcinoma, small cell and non-small cell lung carcinoma, and breast and prostate cancers but, to the best of our knowledge, there is no case associated with endometrial cancer [3, 6]. Moreover, serologic analyses revealed anti-SRP antibodies that are found in 16% of NAM [7]. These antibodies are more frequently found in women (sex ratio of 3.5) [8] and during fall season (possibly caused by a cross-reaction mechanism following a viral infection) [9]. We found one case reporting a patient with lung cancer and anti-SRP antibody [10] raising the question of a possible production of anti-SRP antibodies during neoplastic process.

Our patient initially came to the hospital with chest pain and elevated troponins and CK, but cardiac catheterization was normal. Retrospectively, this episode was most probably the first manifestation of her NAM. Indeed, cardiac involvement can be seen in NAM [1]; the exact incidence is unknown but the electrocardiogram might be abnormal in 50% of cases [8]. Exact consequences of cardiac involvement in NAM are unknown. After this cardiac episode, she received atorvastatin. This treatment could have enhanced the progression of the disease but does not seem to be responsible for it. In fact, the CK level was elevated before the introduction of statins. Furthermore, anti-HMGCR antibodies (a newly discovered autoantibody more frequently found in statin exposed patients) were not found in our patient [11]. Regardless of the etiology of NAM, prednisone is considered as the first-line therapy. Some patients, resistant to the initial therapy, could benefit from steroid-sparing agents such as methotrexate, azathioprine, intravenous immunoglobulin [9], or even rituximab [12]. Our patient accumulated poor prognosis factors [1] including associated malignancy, anti-SRP antibodies, cardiac involvement, and dysphagia. We therefore introduced rapidly a relatively aggressive treatment, which proved, at least so far, well tolerated and associated with marked improvement and the possibility of decreasing considerably the potentially detrimental prednisone doses.

## Figures and Tables

**Table 1:** Laboratory findings.

Variable	Result	Reference range^*∗*^
Creatine kinase (U/L)	10176	<170
Troponin T (ng/L)	1198	<50
Creatine kinase-MB (*μ*g/L)	262.6	<5.0
Aldolase (U/L)	67	1.20–8.80
Aspartate aminotransferase (U/L)	438	10–35
Alanine aminotransferase (U/L)	327	10–35
Lactate dehydrogenase (U/L)	1187	<250
CRP (mg/L)	5.0	<5.0
Creatinine (*μ*mol/L)	33	44–80
Antisignal recognition particle (anti-SRP)	Positive	NA
Anti-HMG coA reductase (anti-HMGCR)	Negative	NA
Antiacetylcholine receptor (anti-AChR)	Negative	NA
Antimuscle specific kinase (anti-MuSK)	Negative	NA
Antivoltage gate calcium channel (anti-VGCC)	Negative	NA

^*∗*^Reference range for nonpregnant adults, with no medical conditions.

## Additional Points

*Learning Points.* (i) NAM is a complex disease; several etiologies can coexist. (ii) Anti-SRP antibodies can also be found in paraneoplastic associated NAM. (iii) Chest pain with elevated troponins can be the first manifestation of NAM. (iv) Early recognition of poor prognosis factors could direct aggressive initial therapy.
